# Abdominal Visceral Infarction in 3 Patients with COVID-19

**DOI:** 10.3201/eid2608.201161

**Published:** 2020-08

**Authors:** Giulia Besutti, Riccardo Bonacini, Valentina Iotti, Giulia Marini, Nicoletta Riva, Giovanni Dolci, Mariarosa Maiorana, Lucia Spaggiari, Filippo Monelli, Guido Ligabue, Giovanni Guaraldi, Paolo Giorgi Rossi, Pierpaolo Pattacini, Marco Massari

**Affiliations:** Azienda Unità Sanitaria Locale di Reggio Emilia–Istituto di Ricovero e Cura a Carattere Scientifico di Reggio Emilia (AUSL–IRCCS), Reggio Emilia, Italy (G. Besutti, R. Bonacini, V. Iotti, G. Marini, N. Riva, G. Dolci, M. Maiorana, L. Spaggiari, P.G. Rossi, P. Pattacini, M. Massari);; University of Modena and Reggio Emilia Clinical and Experimental Medicine PhD Program, Modena, Italy (G. Besutti, M. Maiorana);; University of Modena and Reggio Emilia, Modena (R. Bonacini, G. Dolci, F. Monelli, G. Ligabue, G. Guaraldi)

**Keywords:** COVID-19, SARS-CoV-2, infarction, abdominal viscera, viruses, respiratory infections, coronavirus, 2019 novel coronavirus disease, severe acute respiratory syndrome coronavirus 2, zoonoses

## Abstract

A high incidence of thrombotic events has been reported in patients with coronavirus disease (COVID-19), which is caused by severe acute respiratory syndrome coronavirus-2 (SARS-CoV-2) infection. We report 3 clinical cases of patients in Italy with COVID-19 who developed abdominal viscera infarction, demonstrated by computed tomography.

Frequent thrombotic events, mostly pulmonary embolisms, have been reported in patients with coronavirus disease (COVID-19) (*1*–*4*). We describe 3 cases of COVID-19 complicated by abdominal visceral infarction that occurred in inhabitants of the Emilia Romagna region in northern Italy.

Patient 1, a 54-year-old male former smoker with a history of asthma and quiescent ulcerative colitis not receiving any treatment, was admitted to the emergency department (ED) on February 28, 2020, for syncope. He was discharged after undergoing chest radiography and brain computed tomography (CT), the results of which were unremarkable. He returned to the ED after 5 days for treatment of dyspnea, fatigue, and fever. Blood tests revealed decreased oxygen saturation (94%), increased C-reactive protein (CRP) level (5.38 mg/dL; reference <0.5 mg/dL), and lymphopenia (0.69 × 10^3^ cells/mm^3^; reference range 0.8–4 × 10^3^ cells/mm^3^). Chest CT scan demonstrated bilateral viral pneumonia, and nasopharyngeal and oropharyngeal swab specimens were positive for severe acute respiratory syndrome coronavirus 2 (SARS-CoV-2). He was hospitalized and treated with lopinavir/ritonavir (400/100 mg orally 2×/d), and hydroxychloroquine (200 mg orally 2×/d). He was discharged to home after 3 hospital days, on therapy; no anticoagulant prophylaxis was suggested. He was rehospitalized 6 days after discharge when he developed sharp right flank and lumbar pain, fever, and dysuria. Blood and urine tests revealed neutrophilia (9.9 × 10^3^ cells/mm^3^; reference range 1.6–7.5 × 10^3^ cells/mm^3^), increased lactate dehydrogenase (LDH) (1,507 U/L; reference range 28–378 U/L), increased CRP (1.43 mg/dL), and proteinuria (50 mg/dL). CT scan demonstrated a large right kidney arterial infarction (Figure, panel A). He was treated with low molecular weight heparin (LMWH) (6,000 UI 2×/d) and discharged to home after 4 days.

**Figure:**
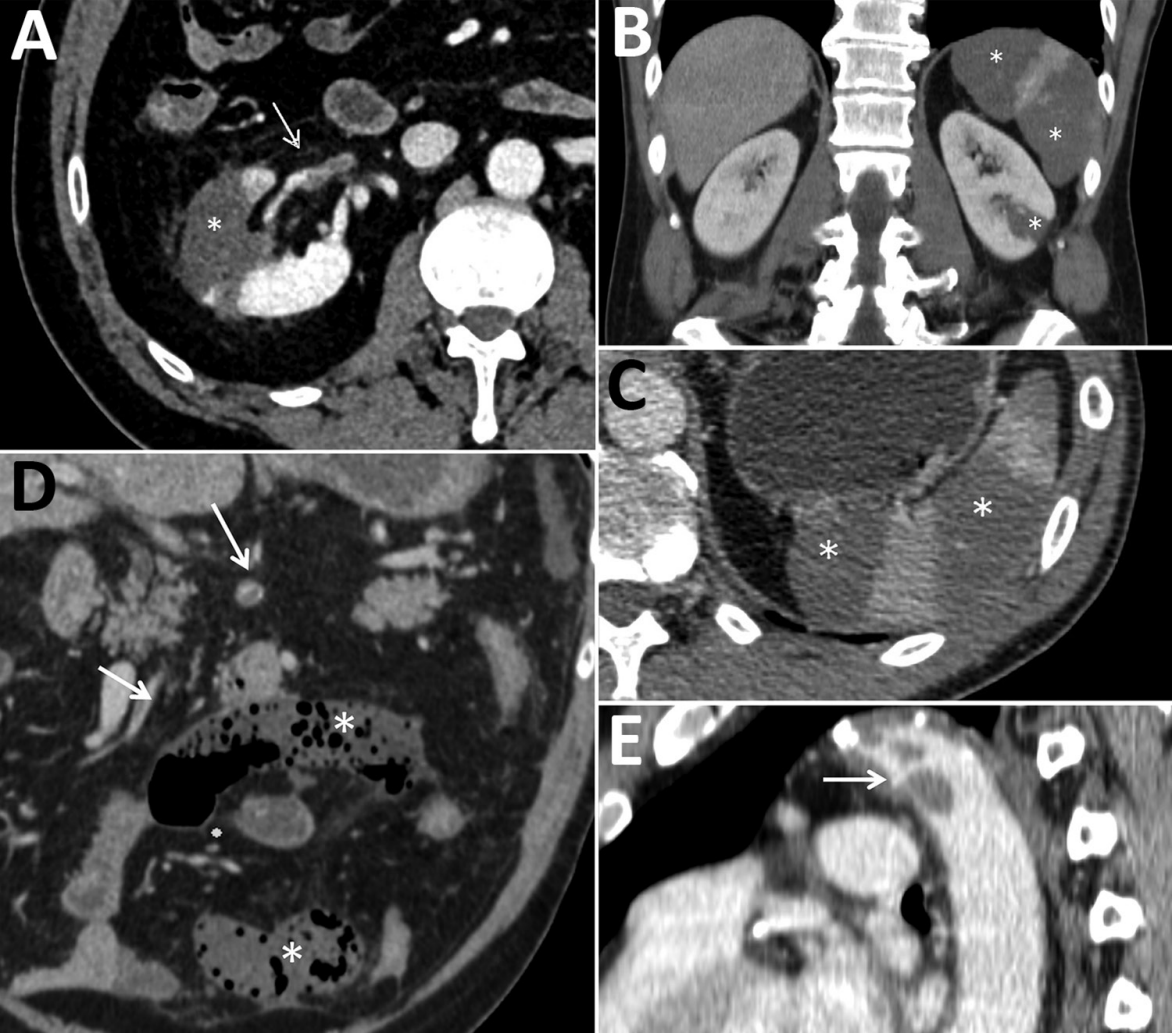
Abdominal contrast-enhanced computed tomography scans of 3 coronavirus disease patients with abdominal visceral infarction, Italy. A) Patient 1 (axial view) showing intraarterial thrombi in the renal artery (arrow) and kidney and splenic infarctions (asterisk), seen as large wedge-shaped hypodense parenchymal areas. B, C) Patient 2 (B, coronal view; C, axial view) showing kidney and splenic infarctions (asterisks), seen as large wedge-shaped hypodense parenchymal areas. D, E) Patient 3 (D, coronal view; E, sagittal view), showing intraarterial thrombi in the superior mesenteric artery and its branches (arrows in D) and thoracic descending aorta (arrow in E), as well as small bowel ischemia (asterisks in D), seen as small bowel loops with decreased or absent wall enhancement. In patients 1 and 2, scans did not show notable signs of atherosclerosis.

Patient 2, a 53-year-old man with hypertension and a history of mitral valve replacement (June 2019), came to the ED on March 11, 2020, with fever, cough, and sore throat. At admission, he had decreased oxygen saturation (94%) and increased CRP (6.99 mg/dL). Chest CT scan demonstrated bilateral viral pneumonia, and nasopharyngeal and oropharyngeal swab specimens were positive for SARS-CoV-2. He was hospitalized and treated with lopinavir/ritonavir (400/100 mg orally 2×/d) and hydroxychloroquine (200 mg orally 2×/d); he also received 2 administrations of tocilizumab (8 mg/kg, an off-label use) on hospital day 3 because his respiratory function was worsening. Because of his previous mitral valve replacement, he was already being treated with antiplatelet prophylaxis with acetylsalicylic acid but not with anticoagulants. On hospital day 6 he reported severe left flank pain; blood tests revealed neutrophilia (11.74 × 10^3^ cells/mm^3^) and increased LDH (932 U/L) and CRP (4.42 mg/dL). CT scan demonstrated large infarcted areas involving the spleen and the left kidney (Figure, panels B,C). He was treated with LMWH (6,000 UI 2×/d) and discharged home after 7 days.

Patient 3, a 72-year-old man with stage 3 kidney failure, hypertension, previous myocardial infarction, and type 2 diabetes, came to the ED on March 25, 2020, with shortness of breath and dry cough. At admission, he had increased CRP (19.3 mg/dL) and high glucose level (1,000 mg/dL; reference <100 mg/dL) with severe metabolic acidosis. Nasopharyngeal and oropharyngeal swab specimens were positive for SARS-CoV-2. He was hospitalized, began antithrombotic prophylaxis with LMWH (4,000 UI 1×/d), and continued secondary prophylaxis with acetylsalicylic acid. He was transferred in the intensive care unit the day after admission; a few hours later, he developed severe abdominal pain. Blood tests revealed neutrophilia (17.69 × 10^3^ cells/mm^3^) and increased LDH (1,510 U/L), CRP (48 mg/dL), and D-dimer (6,910 ng/mL), with normal prothrombin time and activated partial thromboplastin time. Antiphospholipid antibodies were not detected. CT scan demonstrated small bowel ischemia associated with massive splenic infarction (Figure, panels D,E). He underwent resection of the ischemic bowel loop and splenectomy, was treated with heparin in continuous infusion, and was discharged from the ICU 2 days later. As of May 9, he was still hospitalized but his condition was improving.

Between the start of the SARS-CoV-2 outbreak in Reggio Emilia at the end of February and March 24, the province has had 460 hospitalizations in all hospitals. Among these, 2 (0.4%) patients (*1*,*2*) had acute ischemic events involving abdominal viscera; therefore, these events should not be considered too rare. Visceral infarction is probably a clinical manifestation of the prothrombotic state that has been described in patients with COVID-19 (*1*–*6*). Consistently, reports about pathological changes in organs other than the lungs describe parenchymal cells necrosis and small-vessel thrombosis (*7*).

The possibility of abdominal visceral infarction during COVID-19 has major implications in clinical practice. First, when patients with COVID-19 report severe abdominal pain, visceral infarction should be considered in differential diagnosis and taken into account in laboratory and imaging diagnostic workups. Second, this finding should further prompt the scientific community to stress the need to routinely use LMWH in patients with COVID-19 and to open the debate on the appropriate dosage. Finally, the prothrombotic state in patients with COVID-19 may justify therapeutic rather than prophylactic LMWH.

## References

[1] Klok FA, Kruip MJHA, van der Meer NJM, Arbous MS, Gommers DAMPJ, Kant KM, et al. Incidence of thrombotic complications in critically ill ICU patients with COVID-19. Thromb Res. 2020 Apr 10 [Epub ahead of print]. 10.1016/j.thromres.2020.04.013 PMC7146714 32291094

[2] Griffin DO, Jensen A, Khan M, Chin J, Chin K, Saad J, et al. Pulmonary embolism and increased levels of d-dimer in patients with coronavirus disease. Emerg Infect Dis. 2020;26. 10.3201/eid2608.201477 32348233 PMC7392455

[3] Khodamoradi Z, Boogar SS, Shirazi F, Kouhi P. COVID-19 and acute postpartum pulmonary embolism. Emerg Infect Dis. In press 2020. 10.3201/eid2608.201383 PMC7392410 32396506

[4] Helms J, Tacquard C, Severac F, Leonard-Lorant I, Ohana M, Delabranche X, et al.; CRICS TRIGGERSEP Group (Clinical Research in Intensive Care and Sepsis Trial Group for Global Evaluation and Research in Sepsis). High risk of thrombosis in patients with severe SARS-CoV-2 infection: a multicenter prospective cohort study. Intensive Care Med. 2020. 10.1007/s00134-020-06062-x 32367170 PMC7197634

[5] Huang C, Wang Y, Li X, Ren L, Zhao J, Hu Y, et al. Clinical features of patients infected with 2019 novel coronavirus in Wuhan, China. Lancet. 2020;395:497–506. 10.1016/S0140-6736(20)30183-5 31986264 PMC7159299

[6] Zhou F, Yu T, Du R, Fan G, Liu Y, Liu Z, et al. Clinical course and risk factors for mortality of adult inpatients with COVID-19 in Wuhan, China: a retrospective cohort study. Lancet. 2020;395:1054–62. 10.1016/S0140-6736(20)30566-3 32171076 PMC7270627

[7] Yao XH, Li TY, He ZC, Ping YF, Liu HW, Yu SC, et al. A pathological report of three COVID-19 cases by minimally invasive autopsies [in Chinese]. Zhonghua Bing Li Xue Za Zhi. 2020;49:E009. 32172546 10.3760/cma.j.cn112151-20200312-00193

